# Joint Association of Low Vitamin K1 and D Status With First Stroke in General Hypertensive Adults: Results From the China Stroke Primary Prevention Trial (CSPPT)

**DOI:** 10.3389/fneur.2022.881994

**Published:** 2022-05-12

**Authors:** Yaping Wei, Hai Ma, Benjamin Xu, Zhuo Wang, Qiangqiang He, Lishun Liu, Ziyi Zhou, Yun Song, Ping Chen, Jianping Li, Yan Zhang, Guangyun Mao, Binyan Wang, Genfu Tang, Xianhui Qin, Hao Zhang, Xiping Xu, Yong Huo, Huiyuan Guo

**Affiliations:** ^1^Key Laboratory of Precision Nutrition and Food Quality, Department of Nutrition and Health, College of Food Sciences and Nutritional Engineering, China Agricultural University, Beijing, China; ^2^Rongcheng Center for Disease Control and Prevention, Rongcheng, China; ^3^Department of Epidemiology, Harvard T.H. Chan School of Public Health, Boston, MA, United States; ^4^Graduate School at Shenzhen, Tsinghua University, Shenzhen, China; ^5^Shenzhen Evergreen Medical Institute, Shenzhen, China; ^6^Institute for Biomedicine, Anhui Medical University, Hefei, China; ^7^College of Pharmacy, Jinan University, Guangzhou, China; ^8^Department of Cardiology, Peking University First Hospital, Beijing, China; ^9^Department of Preventive Medicine, School of Public Health & Management, Wenzhou Medical University, Wenzhou, China; ^10^National Clinical Research Center for Kidney Disease, State Key Laboratory for Organ Failure Research, Division of Nephrology, Nanfang Hospital, Southern Medical University, Guangzhou, China

**Keywords:** vitamin K1, 25-hydroxyvitamin D, hypertensive adults, nested case-control study, first stroke

## Abstract

**Background:**

Vitamin K plays a role in preventing vascular calcification and may have a synergetic influence with vitamin D on cardiovascular health. However, whether this relationship applies to stroke, especially in a high-risk population of hypertensive individuals, remains unclear. The present study aims to study the joint association of low vitamin K1 and D status with first stroke in general hypertensive adults.

**Methods:**

This study used a nested, case–control design with data from the China Stroke Primary Prevention Trial. The analysis included 604 first total stroke patients and 604 matched controls from a Chinese population with hypertension. Odds ratios (ORs) and 95% confidence intervals were calculated using conditional logistic regression.

**Results:**

There was a non-linear negative association between plasma vitamin K1 and the risk of first total stroke or ischemic stroke in the enalapril-only group. Compared to participants in vitamin K1 quartile 1, a significantly lower risk of total stroke (*OR* = 0.58, 95% CI: 0.36, 0.91, *P* = 0.020) or ischemic stroke (*OR* = 0.34, 95% CI: 0.17, 0.63, *P* < 0.001) was found in participants in vitamin K1 quartile 2-4 in the enalapril-only group. When further divided into four subgroups by 25(OH)D and vitamin K1, a significantly higher risk of total stroke or ischemic stroke was observed in participants with both low vitamin K1 and 25(OH)D compared to those with both high vitamin K1 and 25(OH)D in the enalapril-only group. No increased risk was observed in the groups low in one vitamin only.

**Conclusion:**

Low concentrations of both vitamin K1 and 25(OH)D were associated with increased risk of stroke.

## Introduction

Cardiovascular disease (CVD) is the primary cause of morbidity in the world, leading to more than 17 million deaths per year, with about 6.7 million deaths attributed to stroke (1). Ischemic stroke (IS), caused by the blockage of blood vessels to the brain, accounts for about 80% of the stroke population (2). Stroke is a multifactorial disease that is related to modifiable risk factors such as hypertension, diabetes, and nutrient deficiencies (3). Vitamin K1 is a fat-soluble vitamin mainly obtained from green leafy vegetables and is required for the activation of hepatic coagulation factors. Recent studies have shown that vitamin K1 also plays an important role in bone (4) and cardiovascular health (5). For example, vitamin K is responsible for activating vitamin K-dependent Gla-proteins in extrahepatic tissues, such as matrix Gla-protein (MGP), which has the function of inhibiting vascular calcification (6).

Observational studies and randomized controlled trials have shown that low serum vitamin K1 is significantly associated with coronary artery calcium progression, especially in hypertensive individuals (7, 8). A meta-analysis showed that the presence and severity of coronary artery calcification was related to stroke events during medium and long-term follow-up (9). However, the protective role of vitamin K1 in stroke has not been well-validated in observational studies or clinical trials. A previous study found no association between high dephosphorylated-uncarboxylated Matrix Gla-Protein (dp-ucMGP) levels, reflecting poor vitamin K status, and increased stroke risk in the general population (10). Prior observational studies have also been unable to identify an association between dietary vitamin K1 intake and overall risk of ischemic stroke (11, 12). However, a Mendelian randomization study indicated that a genetic predisposition to higher circulating vitamin K1 levels is associated with an increased risk of large artery atherosclerotic stroke (13). Overall, significant research gaps remain regarding low vitamin K status and stroke, especially among high-risk populations, such as people with hypertension.

Accumulating epidemiological evidence has found that low 25-Hydroxyvitamin D [25(OH)D] levels, which is the general marker of vitamin D status, to be associated with CVD risk factors and increased CVD risk (14). Vitamin D has a potent role in the regulation of the renin–angiotensin aldosterone system, as well as anti-inflammatory, antioxidative (14). It has been reported that 25(OH)D level was inversely related to stroke risk, with a non-linear dose response relationship (15). Previous studies have shown that vitamins D and K have a synergistic effect on cardiovascular health (16, 17). As noted previously, prospective studies of the association between blood concentrations of vitamin K1 and subsequent stroke are sparse, and additionally, the question remains whether a joint association exists between vitamins D and K1, and stroke. This case-control study aimed to investigate the association between plasma vitamin K1 concentrations and the risk of stroke in patients with hypertension, taking into account the possible influence of vitamin D status on this association.

## Materials and Methods

### Study Design and Participants

This study population stems from the China Stroke Primary Prevention Trial (CSPPT) cohort of patients with hypertension. A detailed description of the CSPPT has been provided elsewhere (18). Briefly, CSPPT was a multi-community, randomized, double-blind clinical trial conducted from 2008 to 2013 with 20,702 adults in 32 communities in China. Eligible participants were men and women aged 45–75 years who had hypertension, defined as seated, resting systolic blood pressure (SBP) ≥140 mmHg or diastolic blood pressure (DBP) ≥90 mmHg or who had anti-hypertensive medication at baseline. The major exclusion criteria included history of physician-diagnosed stroke, myocardial infarction (MI), heart failure, post-coronary revascularization, and/or congenital heart disease, and/or current supplementation by folic acid, vitamin B12 or vitamin B6. Eligible participants were randomly assigned in a 1:1 ratio to two treatment group: enalapril-only group and enalapril-folic acid group. In the enalapril-only group, participants received a daily tablet containing 10 mg enalapril only. In the enalapril-folic acid group, participants received a daily tablet containing 10 mg enalapril and 0.8 mg folic acid. Concomitant use of other antihypertensive drugs (mainly calcium channel blockers or diuretics) was allowed during the trial periods, but not B-vitamins.

As shown in Supplementary Figure 1, after a median follow-up of 4.5 years, 637 patients had first stroke in the CSPPT. The present study used a 1:1 matched case–control design. Patients with first stroke were selected as cases (*n* = 635), excluding two cases that could not be matched to a control. Another 635 participants without stroke, matched by baseline age (±1 year), sex, treatment group and study site, served as controls. After excluding participants with missing 25(OH)D and vitamin K1 data, we obtained 604 stroke case-control pairs, of which 484 were ischemic stroke, 118 were hemorrhagic and 2 were undefined case-control pairs.

### Measurement of Vitamin K1 and Vitamin D

A venous blood sample was obtained from each study participant at baseline after overnight fasting. Blood samples were centrifuged and stored at −80°C until analysis. The plasma concentrations of 25(OH)D and vitamin K1 were analyzed to assess the vitamin D and K status of participants. Plasma vitamin K1, 25(OH)D3 and 25(OH)D2 were measured by liquid chromatography with tandem quadrupole mass spectrometry (LC-MS/MS) in a commercial lab (Beijing DIAN Medical Laboratory, China). Total 25(OH)D was used in all analyses and was calculated as the sum of 25(OH)D3 and 25(OH)D2. Season-adjusted vitamin D levels were calculated by adding the residuals from a linear regression model of 25(OH)D by season of blood draw to the overall mean value.

### Outcome Assessment

The stroke outcome in this study was a first non-fatal or fatal stroke (ischemic or hemorrhagic), excluding subarachnoid hemorrhage and silent stroke. All participants underwent brain CT and/or magnetic resonance imaging (MRI). The diagnosis of stroke and stroke subtypes was based on medical records and imaging data that were reviewed by at least two adjudicators who were senior stroke neurologists. Source data for all suspected stroke cases including medical records and imaging data as well as event report forms were submitted to the event adjudication committee for further verification. Stroke etiology in our study was identified by the ICD10 codes of diagnosis. The outcomes were total stroke (ICD9 430,431, 433,434 and 436 or ICD10 I60, I61, I63 and I64), ischemic stroke (ICD9 433 and 434 or ICD10 I63) and hemorrhagic stroke (ICD9 430 and 431 or ICD10 I60 and I61).

### Data Collection and Assessment

Information on age, sex, body mass index (BMI), smoking status, alcohol consumption and other demographic factors, was collected using a standardized questionnaire. Current smokers were defined as smoking at least 1 cigarette per day or smoking >18 packs for the past 12 months; ex-smokers were those who had not smoked in at least 12 months before enrollment, and all others were defined as never smokers. Current alcohol drinkers were defined as individuals who consumed ≥3 drinks per week over the past year; former drinkers were those who had quit drinking for more than 1 year, and all others were defined as never alcohol drinkers (19, 20). Fasting venous blood samples were also collected at enrollment and at the exit visit. Seated blood pressure was measured by trained research staff after participants had rested for 10 min. Baseline plasma fasting glucose, serum fasting lipids, homocysteine and fasting serum total calcium (arsenazo-III method) levels were measured using automatic clinical analyzers (Beckman Coulter). Serum folate levels were measured in a commercial laboratory using a chemiluminescent immunoassay (New Industrial). The MTHFRC677T (rs1801133) polymorphism of methylenetetrahydrofolate reductase (MTHFR), the main regulatory enzyme for folate metabolism, was detected using the Taq Man assay.

### Statistical Analysis

Normally distributed continuous variables were presented as mean ± standard deviation and compared using *t*-tests. Non-normally distributed continuous variables were presented as median (75th percentile−25th percentile) and compared using rank-sum tests. Categorical variables were presented as number (percentage) and were compared using chi-square tests. All covariates had <5% total missing data. Missing values of continuous variables were replaced by the median and missing values of categorical variables were replaced by a large proportion of values. Logarithmic transformation was performed to normalize the distribution of vitamin K1. Multivariate conditional logistic regression analysis was performed to evaluate the association between vitamin K1 and stroke. All of the potential confounders in the univariate analyses were included in a stepwise conditional logistic regression analysis as the adjustment variable-selection process. The inclusion criterion for the stepwise regression analysis was *P* = 0.3 and the elimination criterion was *P* = 0.2. The adjusted variables in the final model included: body mass index (BMI, kg/m^2^), baseline SBP (mmHg), time-averaged SBP and DBP, baseline fasting blood glucose (mmol/L), total cholesterol (mmol/L), triglycerides (mmol/L), serum folate (ng/mL), antihypertensive medication usage at baseline (yes vs. no), smoking status (ever vs. never) and antiplatelet drug usage at baseline (yes vs. no). Potential interactions were examined by including the interaction terms in the logistic regression models. A two tailed *P* < 0.05 was considered statistically significant in all analyses. R software, version 3.2.5 (http://www.R-project.org/) was used for all statistical analyses.

## Results

### Study Participants and Baseline Characteristics

Individuals with missing vitamin K1 and/or D data and unpaired cases or controls were excluded from the analysis. The final analyses included 604 total stroke cases matched with 604 controls (Supplementary Figure 1). A total of 672 (55.6%) participants were in the enalapril-only group and 536 (44.4%) participants were in the enalapril-folic acid group. The mean age of the study population was 62.2 years, and 46.9% were male. Baseline characteristics of total participants by case-control status and by treatment group are presented in Table 1. In the enalapril-only group, stroke cases had high total cholesterol and high blood pressure at baseline and during follow-up. In the enalapril-folic acid group, stroke cases had high body mass index and high blood pressure at baseline and during follow-up.

**Table 1:** Characteristics of the participants by case-control status and by treatment group^a^.

**Variables**	**Enalapril-only group**			**Enalapril-folic acid group**		
	**Cases (*n* = 336)**	**Controls (*n* = 336)**	***P***	**Cases (*n* = 268)**	**Controls (*n* = 268)**	***P***
Age, year	62.1 (56.9,67.8)	62.1 (56.9,67.9)	0.995	62.9 (56.6,68.1)	62.9 (56.7,67.9)	0.998
Male (%)	166 (49.4)	166 (49.4)	1.000	117 (43.7)	117 (43.7)	1.000
Body mass index, kg/m^2^	24.9 ± 3.7	24.8 ± 3.5	0.814	25.5 ± 3.6	24.8 ± 3.3	0.016
Baseline SBP, mmHg	176.0 (160.7, 190.0)	162.7 (153.3, 179.3)	<0.001	171.7 (159.7, 189.3)	164.0 (156.2, 179.3)	<0.001
Baseline DBP, mmHg	98.0 (88.7, 105.7)	92.0 (85.3, 100.0)	<0.001	98.0 (89.3, 102.7)	92.3 (85.7, 100.7)	0.006
Average SBP, mmHg	147.1 (138.6, 158.7)	137.9 (131.7, 145.5)	<0.001	147.1 (138.9, 159.2)	137.9 (132.0, 146.4)	<0.001
Average DBP, mmHg	85.9 (80.8, 91.0)	81.7 (78.0, 86.1)	<0.001	86.0 (80.6, 92.6)	81.3 (77.5, 86.5)	<0.001
Laboratory results						
Total cholesterol, mmol/L	5.7 (5.0, 6.5)	5.5 (4.8, 6.3)	0.004	5.6 (4.8, 6.3)	5.6 (4.7, 6.4)	0.814
Triglycerides, mmol/L	1.5 (1.1, 2.0)	1.3 (1.0, 1.9)	0.231	1.4 (1.1, 1.9)	1.4 (1.0, 1.8)	0.669
HDL-C, mmol/L	1.3 (1.1, 1.5)	1.3 (1.1, 1.5)	0.479	1.3 (1.1, 1.6)	1.3 (1.1, 1.6)	0.134
Fasting blood glucose, mmol/L	5.6 (5.1, 6.6)	5.5 (5.1, 6.3)	0.227	5.5 (5.0, 6.2)	5.5 (4.9, 6.1)	0.578
Homocysteine, μmol/L	13.4 (11.2, 16.7)	12.9 (10.8, 15.6)	0.070	13.2 (10.9, 16.7)	13.0 (10.9, 15.9)	0.522
Folate, ng/mL	7.8 (5.3, 10.0)	7.8 (5.4, 9.8)	0.644	7.5 (5.5, 10.1)	8.3 (5.6, 10.5)	0.111
Uric acid, μmol/L	292.0 (243.0, 344.5)	293.0 (241.5, 352.0)	0.623	290.0 (248.5, 350.0)	289.5 (248.0, 348.0)	0.83
25(OH)D, ng/mL	19.4 (15.4, 24.4)	19.3 (14.8, 24.3)	0.601	18.6 (14.5, 23.4)	19.5 (14.3, 24.2)	0.346
Vitamin K1, ng/mL	0.5 (0.2, 1.0)	0.5 (0.3, 1.1)	0.398	0.5 (0.3, 1.0)	0.6 (0.3, 1.2)	0.646
Calcium, mg/dL	9.7 (9.4, 10.1)	9.7 (9.3, 10.0)	0.304	9.7 (9.3, 10.0)	9.7 (9.4, 10.1)	0.181
Hypotensive drug usage	169 (50.3)	160 (47.6)	0.487	143 (53.4)	123 (45.9)	0.084
Antiplatelet drug usage	10 (3.0)	14 (4.2)	0.406	8 (3.0)	7 (2.6)	0.793
Smoking status (%)			0.803			0.076
No	210 (62.5)	212 (63.1)		162 (60.4)	186 (69.4)	
Current	93 (27.7)	87 (25.9)		82 (30.6)	60 (22.4)	
Former	33 (9.8)	37 (11.0)		24 (9.0)	22 (8.2)	
Alcohol drinking (%)			0.687			0.453
No	218 (64.9)	220 (65.5)		181 (67.5)	191 (71.3)	
Current	94 (28.0)	87 (25.9)		58 (21.6)	56 (20.9)	
Former	24 (7.1)	29 (8.6)		29 (10.8)	21 (7.8)	
Study site			1.000			1.000
Anqing	57 (17.0)	57 (17.0)		57 (21.3)	57 (21.3)	
Lianyungang	279 (83.0)	279 (83.0)		211 (78.7)	211 (78.7)	
MTHFR C677T (%)			0.424			0.634
CC	94 (28.0)	92 (27.4)		62 (23.1)	68 (25.4)	
CT	149 (44.3)	164 (48.8)		138 (51.5)	127 (47.4)	
TT	93 (27.7)	80 (23.8)		68 (25.4)	73 (27.2)	

a *DBP, diastolic blood pressure; HDL-C, high-density lipoprotein cholesterol; MTHFR, methylenetetrahydrofolate reductase; SBP, systolic blood pressure; 25(OH)D, 25-hydroxyvitamin D*.

### Baseline Plasma Vitamin K1 and the Risk of First Stroke

Table 2 presents the results of the independent associations between vitamin K1 levels and the risk of stroke (total and subtypes) adjusted by potential confounding factors. For the total population, no significant results were seen between the risk of total stroke and its subtypes. The participants in the second, third and fourth quartiles of vitamin K1 showed a decreased odds of risk of ischemic stroke compared to those in quartile 1 in the unadjusted and adjusted models, although these associations did not reach statistical significance.

**Table 2:** The relationship of baseline plasma vitamin K1 with the risk of stroke (total and subtypes)^a^.

**Vitamin K1, ng/mL**	**N**	**Cases (%)**	**Crude model**		**Adjusted model**	
			**OR (95%CI)**	***P***	**OR (95%CI)**	***P***
First total stroke (*n* = 1208)^b^						
Q1 (<0.26)	302	159 (52.60)	1 (Reference)		1 (Reference)	
Q2 (0.26- <0.54)	302	144 (47.70)	0.79 (0.56, 1.12)	0.183	0.93 (0.61, 1.41)	0.735
Q3 (0.54- <1.08)	302	160 (53.00)	0.94 (0.63, 1.39)	0.740	1.17 (0.72, 1.91)	0.515
Q4 (≥1.08)	302	141 (46.70)	0.70 (0.46, 1.06)	0.096	0.92 (0.55, 1.56)	0.765
Categories						
Q1 (<0.26)	302	159 (52.60)	1 (Reference)		1 (Reference)	
Q2-Q4 (≥0.26)	906	445 (49.10)	0.81 (0.58, 1.11)	0.189	0.98 (0.66, 1.46)	0.921
Ischemic stroke (*n* = 968)^b^						
Q1 (<0.28)	241	130 (53.90)	1 (Reference)		1 (Reference)	
Q2 (0.28– <0.58)	243	113 (46.50)	0.71 (0.49, 1.04)	0.081	0.70 (0.44, 1.11)	0.131
Q3 (0.58– <1.13)	242	124 (51.20)	0.82 (0.53, 1.27)	0.379	0.73 (0.43, 1.24)	0.239
Q4 (≥1.13)	242	117 (48.30)	0.72 (0.46, 1.13)	0.155	0.71 (0.40, 1.25)	0.238
Categories						
Q1 (<0.28)	241	130 (53.90)	1 (Reference)		1 (Reference)	
Q2-Q4 (≥0.28)	727	354 (48.70)	0.740 (0.52, 1.05)	0.093	0.71 (0.46, 1.09)	0.121
Hemorrhagic stroke (*n* = 336)^c^						
Q1 (<0.22)	59	29 (49.20)	1 (Reference)		1 (Reference)	
Q2 (0.22– <0.40)	59	29 (49.20)	0.98 (0.43, 2.24)	0.969	1.25 (0.46, 3.48)	0.662
Q3 (0.40– <0.85)	59	36 (61.00)	1.51 (0.61, 3.81)	0.377	4.92 (1.39, 20.25)	0.019
Q4 (≥0.85)	59	24 (40.70)	0.49 (0.15, 1.49)	0.217	1.60 (0.30, 8.91)	0.585
Categories						
Q1 (<0.22)	59	29 (49.20)	1 (Reference)		1 (Reference)	
Q2-Q4 (≥0.22)	177	89 (50.30)	1.08 (0.50, 2.32)	0.848	1.69 (0.66, 4.50)	0.277

a *CI, confidence interval; OR, odds ratio; Q1, quartile 1; Q2, quartile 2; Q3, quartile 3; Q4, quartile 4*.

b *Conditional logistic regression models were used. Adjusted models included the following covariables: body mass index, baseline systolic blood pressure, time-averaged systolic blood pressure and diastolic blood pressure during treatment, baseline fasting blood glucose, total cholesterol, triglycerides, folate, antihypertensive medications, smoking status and antiplatelet drug usage at baseline*.

c *Conditional logistic regression models were used. Adjusted models included the following covariables: time-averaged systolic blood pressure and diastolic blood pressure during treatment and antihypertensive treatment at baseline*.

Subgroup analyses were performed based on the potential confounding factors (Supplementary Figures 2, 3). A significantly stronger inverse association between plasma vitamin K1 (quartile 2–4 vs. quartile 1) and total stroke risk or ischemic stroke risk was observed in the enalapril-only group, while the association was slightly positive for participants in the enalapril-folic acid group. The interaction was significant (*P* = 0.010 for total stroke and *P* = 0.024 for ischemic stroke). Moreover, a trend of lower risk of total stroke or ischemic stroke with higher concentrations of vitamin K1 was observed in participants with serum calcium levels ≥9.7 mg/dL but not in participants with low serum calcium levels. Tests for interaction were significant (*P* = 0.022 for total stroke and *P* = 0.046 for ischemic stroke).

Given that folic acid treatment might affect the association between vitamin K1 and stroke risk, the possible effect of treatment group on the vitamin K1—first stroke association was further investigated. Overall, there was a non-linear, negative association between plasma vitamin K1 and the risk of first total stroke or ischemic stroke in the enalapril-only group (Table 3). Compared to participants with vitamin K1 in quartile 1, a significantly lower risk of total stroke (*OR* = 0.58, 95% CI: 0.36, 0.91, *P* = 0.020) or ischemic stroke (*OR* = 0.34, 95% CI: 0.17, 0.63, *P* < 0.001) was found in participants with vitamin K1 in quartile 2–4 in the enalapril-only group. However, in the enalapril-folic acid group, a significantly higher first total stroke risk, but not ischemic stroke risk, was found in participants with vitamin K1 in quartile 2–4 (*OR* = 2.43; 95% CI: 1.31, 4.66, *P* = 0.006), compared to those with vitamin K1 in quartile 1. Furthermore, there was no significant association between plasma vitamin K1 and first hemorrhagic stroke in either treatment group.

**Table 3:** Baseline plasma vitamin K1 and the risk of first stroke (total and subtypes) by treatment group^a^.

**Vitamin K1, ng/mL^**b**^**	**Enalapril-only group**					**Enalapril-folic acid group**				
	**Case/Control**	**Crude model**		**Adjusted model**		**Case/Control**	**Crude model**		**Adjusted model**	
		**OR (95% CI)**	***P***	**OR (95% CI)**	***P***		**OR (95% CI)**	***P***	**OR (95% CI)**	***P***
Total stroke^b^										
Q1	97/76	1 (Reference)		1 (Reference)		62/67	1 (Reference)		1 (Reference)	
Q2	72/95	0.53 (0.32, 0.86)	0.011	0.41 (0.22, 0.75)	0.004	72/63	1.24 (0.75, 2.08)	0.404	2.42 (1.27, 4.77)	0.009
Q3	86/82	0.68 (0.39, 1.17)	0.168	0.52 (0.26, 1.02)	0.059	74/60	1.32 (0.74, 2.37)	0.344	3.04 (1.43, 6.69)	0.005
Q4	81/83	0.63 (0.35, 1.12)	0.121	0.61 (0.29, 1.28)	0.197	60/78	0.75 (0.40, 1.41)	0.376	1.61 (0.71, 3.71)	0.255
Categories										
Q1	97/76	1 (Reference)		1 (Reference)		62/67	1 (Reference)		1 (Reference)	
Q2–Q4	239/260	0.59 (0.37, 0.93)	0.025	0.58 (0.36, 0.91)	0.020	206/201	1.16 (0.72, 1.87)	0.548	2.43 (1.31, 4.66)	0.006
Ischemic stroke^b^										
Q1	82/60	1 (Reference)		1 (Reference)		48/51	1.00 (1.00, 1.00)	Ref.	1 (Reference)	
Q2	62/82	0.49 (0.29, 0.82)	0.007	0.31 (0.16, 0.61)	<0.001	51/48	1.14 (0.64, 2.02)	0.661	1.77 (0.87, 3.66)	0.117
Q3	65/67	0.58 (0.31, 1.04)	0.069	0.33 (0.15, 0.69)	0.004	59/51	1.24 (0.65, 2.39)	0.512	1.76 (0.76, 4.15)	0.189
Q4	65/65	0.60 (0.33, 1.10)	0.101	0.43 (0.19, 0.94)	0.037	52/60	0.89 (0.44, 1.79)	0.732	1.32 (0.54, 3.22)	0.545
Categories										
Q1	82/60	1 (Reference)		1 (Reference)		48/51	1 (Reference)		1 (Reference)	
Q2–Q4	192/214	0.53 (0.32, 0.86)	0.011	0.34 (0.17, 0.63)	<0.001	162/159	1.12 (0.66, 1.90)	0.686	1.69 (0.87, 3.37)	0.129
Hemorrhagic stroke^c^										
Q1	17/15	1 (Reference)		1 (Reference)		12/15	1 (Reference)		1 (Reference)	
Q2	12/14	0.71 (0.20, 2.30)	0.567	0.45 (0.09, 1.94)	0.292	17/16	1.22 (0.38, 4.12)	0.740	1.17 (0.30, 4.86)	0.825
Q3	16/12	0.90 (0.19, 4.00)	0.886	1.39 (0.20, 11.21)	0.740	20/11	2.35 (0.72, 8.67)	0.171	4.01 (0.90, 22.42)	0.085
Q4	16/20	0.49 (0.08, 2.55)	0.398	0.71 (0.07, 8.05)	0.772	8/15	0.33 (0.04, 1.79)	0.227	0.49 (0.04, 4.38)	0.541
Categories										
Q1	17/15	1 (Reference)		1 (Reference)		12/15	1 (Reference)		1 (Reference)	
Q2–Q4	44/46	0.71 (0.21, 2.24)	0.566	054 (0.12, 2.14)	0.380	45/42	1.50 (0.54, 4.47)	0.442	1.91 (0.58, 7.14)	0.303

a *CI, confidence interval; OR, odds ratio; Q1, quartile 1; Q2, quartile 2; Q3, quartile 3; Q4, quartile 4; for total stroke: Q1 (<0.26), Q2 (0.26- <0.54), Q3 (0.54- <1.08), Q4 (≥1.08); for ischemic stroke: Q1 (<0.28), Q2 (0.28- <0.58), Q3 (0.58- <1.13), Q4 (≥1.13); for hemorrhagic stroke: Q1 (<0.22), Q2 (0.22- <0.40), Q3 (0.40- <0.85), Q4 (≥0.85)*.

b *Conditional logistic regression models were used. Adjusted models included the following covariables: body mass index, baseline systolic blood pressure, time-averaged systolic blood pressure and diastolic blood pressure during treatment, baseline fasting blood glucose, total cholesterol, triglycerides, folate, antihypertensive treatment, smoking status and antiplatelet drug usage at baseline*.

c *Conditional logistic regression models were used. Adjusted models included the following covariables: time-averaged systolic blood pressure and diastolic blood pressure during treatment and antihypertensive treatment at baseline*.

### Joint Associations Between Plasma Vitamin K1, 25(OH)D and the Risk of Stroke

**Plasma** 25(OH)D and vitamin K1 were divided into categorical variables using clinical cut points for 25(OH)D (20 ng/mL) and the first quartile value for vitamin K1. Using high levels of both plasma 25(OH)D and vitamin K1 as the reference, only the combination of low 25(OH)D and low vitamin K1 was associated with increased risk of total stroke (*OR* = 2.36, 95%CI: 1.17,4.90, *P* = 0.018) in the enalapril-only group (Table 4). The test of interaction was significant (*P* = 0.040). The combination of low 25(OH)D and low vitamin K1 was associated with increased risk of ischemic stroke (*OR* = 3.81, 95% CI: 1.70, 9.00, *P* = 0.002) in the enalapril-only group. The test of interaction was significant (*P* = 0.036). No increased risk was observed in the groups low in one vitamin only. In addition, no significant association between the combination of low 25(OH)D and low vitamin K1 with the risk of first total stroke or ischemic stroke was found in the enalapril-folic acid group. Moreover, the combination of low 25(OH)D and low vitamin K1 was not associated with the risk of hemorrhagic stroke, regardless of the group of treatment.

**Table 4:** The association of plasma vitamin K1 and 25 hydroxyvitamin D [25(OH)D] status with stroke (total and subtypes)^a^.

**25(OH)D, ng/ml**	**Vitamin K1, ng/mL**	**N**	**Cases (%)**	**Crude model**		**Adjusted model**	
				**OR (95%CI)**	***P***	**OR (95%CI)**	***P***
Total stroke^b^
Enalapril-only group
≥20	≥0.26	244	121 (49.60)	1 (Reference)		1 (Reference)	
≥20	<0.26	71	36 (50.70)	1.27 (0.68, 2.41)	0.455	1.18 (0.52, 2.68)	0.697
<20	≥0.26	255	118 (46.30)	0.87 (0.58, 1.28)	0.468	0.74 (0.45, 1.22)	0.243
<20	<0.26	102	61 (59.80)	1.85 (1.05, 3.32)	0.035	2.36 (1.17, 4.90)	0.018
P for interaction				0.174		0.040
Enalapril-folic acid group
≥20	≥0.26	189	90 (47.60)	1 (Reference)		1 (Reference)	
≥20	<0.26	49	24 (49.00)	1.03 (0.51, 2.06)	0.94	0.38 (0.15, 0.91)	0.033
<20	≥0.26	218	116 (53.20)	1.31 (0.86, 2.01)	0.218	1.16 (0.71, 1.92)	0.555
<20	<0.26	80	38 (47.50)	0.97 (0.54, 1.74)	0.917	0.49 (0.22, 1.04)	0.068
P for interaction				0.447		0.830
Ischemic stroke^b^
Enalapril-only group
≥20	≥0.28	189	94 (49.70)	1 (Reference)		1 (Reference)	
≥20	<0.28	61	31 (50.80)	1.21 (0.61, 2.42)	0.592	1.44 (0.58, 3.64)	0.434
<20	≥0.28	217	98 (45.20)	0.81 (0.52, 1.25)	0.340	0.81 (0.46, 1.42)	0.465
<20	<0.28	81	51 (63.00)	2.12 (1.13, 4.12)	0.022	3.81 (1.70, 9.00)	0.002
P for interaction				0.079		0.036
Enalapril-folic acid group
≥20	≥0.28	148	70 (47.30)	1 (Reference)		1 (Reference)	
≥20	<0.28	41	21 (51.20)	1.17 (0.54, 2.58)	0.688	0.61 (0.23, 1.60)	0.313
<20	≥0.28	173	92 (53.20)	1.33 (0.82, 2.18)	0.255	1.15 (0.64, 2.08)	0.635
<20	<0.28	58	27 (46.60)	0.98 (0.5, 1.94)	0.959	0.67 (0.28, 1.59)	0.369
P for interaction				0.340		0.945
Hemorrhagic stroke^c^
Enalapril-only group
≥20	≥0.28	52	24 (46.20)	1 (Reference)		1 (Reference)	
≥20	<0.28	13	8 (61.50)	2.15 (0.51, 10.53)	0.309	3.34 (0.49, 29.25)	0.232
<20	≥0.28	38	20 (52.60)	1.38 (0.54, 3.64)	0.506	0.78 (0.18, 3.29)	0.725
<20	<0.28	19	9 (47.40)	1.31 (0.34, 5.28)	0.693	1.08 (0.19, 6.08)	0.933
P for interaction				0.332		0.496
Enalapril-folic acid group
≥20	≥0.28	40	19 (47.50)	1 (Reference)		1 (Reference)	
≥20	<0.28	8	4 (50.00)	0.96 (0.21, 4.34)	0.960	0.48 (0.07, 2.90)	0.425
<20	≥0.28	47	26 (55.30)	1.36 (0.61, 3.11)	0.454	1.17 (0.41, 3.44)	0.766
<20	<0.28	19	8 (42.10)	0.63 (0.16, 2.27)	0.491	0.62 (0.13, 2.77)	0.540
P for interaction				0.441		0.923

a *CI, confidence interval; OR, odds ratio; 25(OH)D, 25-hydroxyvitamin D*.

b *Conditional logistic regression models were used. Adjusted models included the following covariables: body mass index, baseline systolic blood pressure, time-averaged systolic blood pressure and diastolic blood pressure during treatment, baseline fasting blood glucose, total cholesterol, triglycerides, folate, antihypertensive treatment, smoking status and antiplatelet drug usage at baseline*.

c *Conditional logistic regression models were used. Adjusted models included the following covariables: time-averaged systolic blood pressure and diastolic blood pressure during treatment and antihypertensive treatment at baseline*.

## Discussion

In this population-based case-control study, we reported on the association of plasma vitamin K1 with stroke in hypertensive patients. There was a significant interactive effect of vitamin K1 and folic acid treatment on total stroke or ischemic stroke. A combination of low vitamin D and K status was statistically associated with increased risk of total stroke or ischemic stroke, especially in those participants in the enalapril-only group.

A large body of evidence supports the beneficial effects of vitamin K1 on musculoskeletal health as well as cardiovascular health. Low nutritional intake and bioavailability of vitamin K appears to be a plausible risk factor for stroke, *via* the importance of vitamin K in the maturation of MGP (one of the vitamin K–dependent proteins) as an inhibitor of tissue calcification (21). Two possible explanations for the results of our study are that the vitamin K concentrations were relatively low and participants were patients with hypertension. There is increasing evidence that the role of vitamin K in CVD may be particularly important in certain high-risk subgroups. Results from the Health, Aging, and Body Composition Study suggest that low plasma vitamin K1 is associated with a higher CVD risk in older adults treated for hypertension (22). Notably, treatment intervention in our study might be a significant modifier: a stronger association was found in participants treated with enalapril alone. Since adults with hypertension in our study were more likely to have lower serum folate than in US (medium 7.82 vs. 11.5 ng/mL) (23). Folic acid has a potent antioxidant and antithrombotic effect in the prevention of cardiovascular disease (24). Therefore, we hypothesize that folic acid treatment may have possibly attenuated the relationships between low vitamin K1 and high stroke risk. It has previously been reported that the combined use of enalapril and folic acid can significantly reduce the risk of first stroke compared with enalapril alone (18).

A meta-analysis reported an association between vitamin D deficiency and stroke (15). Researchers have also presented a potential physiological role of vitamin D in regulating vascular calcification (25). To our knowledge, the associations between vitamin D and K concentrations on stroke have only been studied in isolation. While prior studies have examined vitamins D and K together, they did not focus on stroke. Previous prospective studies have reported substantial interactions for the combined effect of insufficient vitamin D and K status in terms of blood pressure (26) and aortic stiffness (27). The first study of a double-blind placebo-controlled trial to explore the combined effect of vitamin D and K supplementation on cardiovascular health revealed that supplementation of vitamin K1, vitamin D, and minerals was superior to vitamin D and minerals alone in preventing a decrease of elastic properties of the carotid artery, over a 3-year follow-up (28). A recent, large, prospective cohort study in the Netherlands showed that the combined association of low vitamin D and K status with mortality risk was greater than the sum of low vitamin D and K status alone (29). Another clinical study also demonstrated that combined vitamin D and K deficiency is highly prevalent in kidney transplant recipients and is associated with increased mortality and graft failure risk compared with high vitamin D and K status (30). Our results corroborate the findings of this trial: combined low vitamin D and vitamin K status was associated with a higher risk of stroke compared to those with high vitamin D and K status.

We have not yet discovered the exact physiological mechanisms explaining the joint association between vitamin K, vitamin D and stroke. However, some potential overlaps in their action exist. Both vitamin D and K can stimulate the γ-carboxylase system of vitamin K–dependent proteins. Two of these proteins are osteocalcin and MGP, which play a role in cardiovascular health (31, 32). Osteocalcin has been shown to regulate bone mineralization and calcium homeostasis, with the potential to prevent calcium build-up in soft tissues, thereby preventing vascular calcification (33). Similarly, MGP limits calcium incorporation into the extracellular matrix of soft tissues, thus acting as a potent inhibitor of soft tissue calcification. The MGP gene contains a vitamin D (calcitriol) responsive element in its promoter region (34). Circulating vitamin D has been shown to upregulate MGP expression in vascular smooth muscle cells of rats (35). It has also been demonstrated that vitamin K promotes 1,25-dihydroxyvitamin D–stimulated osteocalcin accumulation and mineralization (36). In addition, vitamin D and K have prominent anti-inflammatory effects (37), which is a modifiable risk factor for cardiovascular and cerebrovascular diseases. Thus, vitamins K and D can mutually enhance each other's physiological roles and our results provide a plausible clinical correlate to these experimental findings.

The main strength of our study is that it was conducted on a large population-based cohort, with well-designed quality assurance and quality control throughout. We assessed the joint associations of 25(OH)D and vitamin K1 in relation to first stroke in a population free of baseline stroke through a 1:1 case-control matching design, minimizing potential bias and misclassification. Despite these advantages, our study has several limitations. First, our study faces the same limitations as all case-control studies. A large randomized interventional trial that comparts a placebo, each vitamin separately, and both vitamins combined, would fully elucidate the real clinical impact of vitamin K-D in terms of stroke benefit. Second, although we adjusted for multiple confounding factors, our results may have been influenced by other unknown factors that should be better accounted for in future studies. High vitamin K1 and D levels might reflect healthy dietary and lifestyle patterns, rather than the nutrient itself, however, we were not able to exclude the effects of dietary/lifestyle habits on the results, as these data were not available in our study. Third, given the specificity of our study population (elderly Chinese with hypertension), findings from this study cannot be readily generalized to other populations. Furthermore, since our study population only included Han Chinese, further confirmatory studies in other ethnic groups are needed. Finally, expression levels of MGP were not measured in our study and further studies are needed to determine whether vitamin K is associated with stroke through this pathway.

In conclusion, in the present study, we observed that the simultaneous presence of low vitamin K and D status was associated with a significantly higher risk of ischemic stroke in a hypertensive, population-based sample. We speculate that this phenomenon reflects an overlap in the pleiotropic functions of both vitamins, resulting in a synergistic magnification of disease risk when both are insufficient. Larger, prospective interventional studies are needed to verify these findings.

## Data Availability Statement

The raw data supporting the conclusions of this article will be made available by the authors, without undue reservation.

## Ethics Statement

The studies involving human participants were reviewed and approved by Ethics Committee of the Institute of Biomedicine, Anhui Medical University, Hefei, China (FWA assurance number: FWA00001263). The patients/participants provided their written informed consent to participate in this study.

## Author Contributions

YW and HM analyzed the data and wrote the manuscript. BX, ZW, ZZ, GM, and HZ helped with copyediting. LL and PC audited the data. YS, JL, YZ, YH, BW, GT, XQ, and XX conducted research. YH and HG had primary responsibility for the final content of the manuscript. All authors read and approved the final manuscript.

## Funding

This work was supported by the National Key Research and Development Program [2016YFE0205400, 2018ZX09739010, and 2018ZX09301034003], the Department of Science and Technology of Guangdong Province [2020B121202010], the Science and Technology Planning Project of Guangzhou, China [201707020010], the Science, Technology and Innovation Committee of Shenzhen [GJHS20170314114526143 and JSGG20180703155802047], the Economic, Trade and Information Commission of Shenzhen Municipality [20170505161556110, 20170505160926390, and 201705051617070], and the 111 Project from the Education Ministry of China [No. B18053].

## Conflict of Interest

The authors declare that the research was conducted in the absence of any commercial or financial relationships that could be construed as a potential conflict of interest. The reviewer MH declared a past co-authorship with several of the authors YH and XQ to the handling editor.

## Publisher's Note

All claims expressed in this article are solely those of the authors and do not necessarily represent those of their affiliated organizations, or those of the publisher, the editors and the reviewers. Any product that may be evaluated in this article, or claim that may be made by its manufacturer, is not guaranteed or endorsed by the publisher.
