# Supplementary material for: Joint Association of Low Vitamin K1 and D Status With First Stroke in General Hypertensive Adults: Results From the China Stroke Primary Prevention Trial (CSPPT)
Source: Front Neurol. 2022 May 12;13:881994. doi: 10.3389/fneur.2022.881994 (PMC9135055; doi:10.3389/fneur.2022.881994)
Supplement: Supplementary file 1 [file Data_Sheet_1.docx]

**Joint association of low vitamin K1 and D status with first stroke in general hypertensive adults: results from the China Stroke Primary Prevention Trial (CSPPT)**

**Supplemental materials**

| 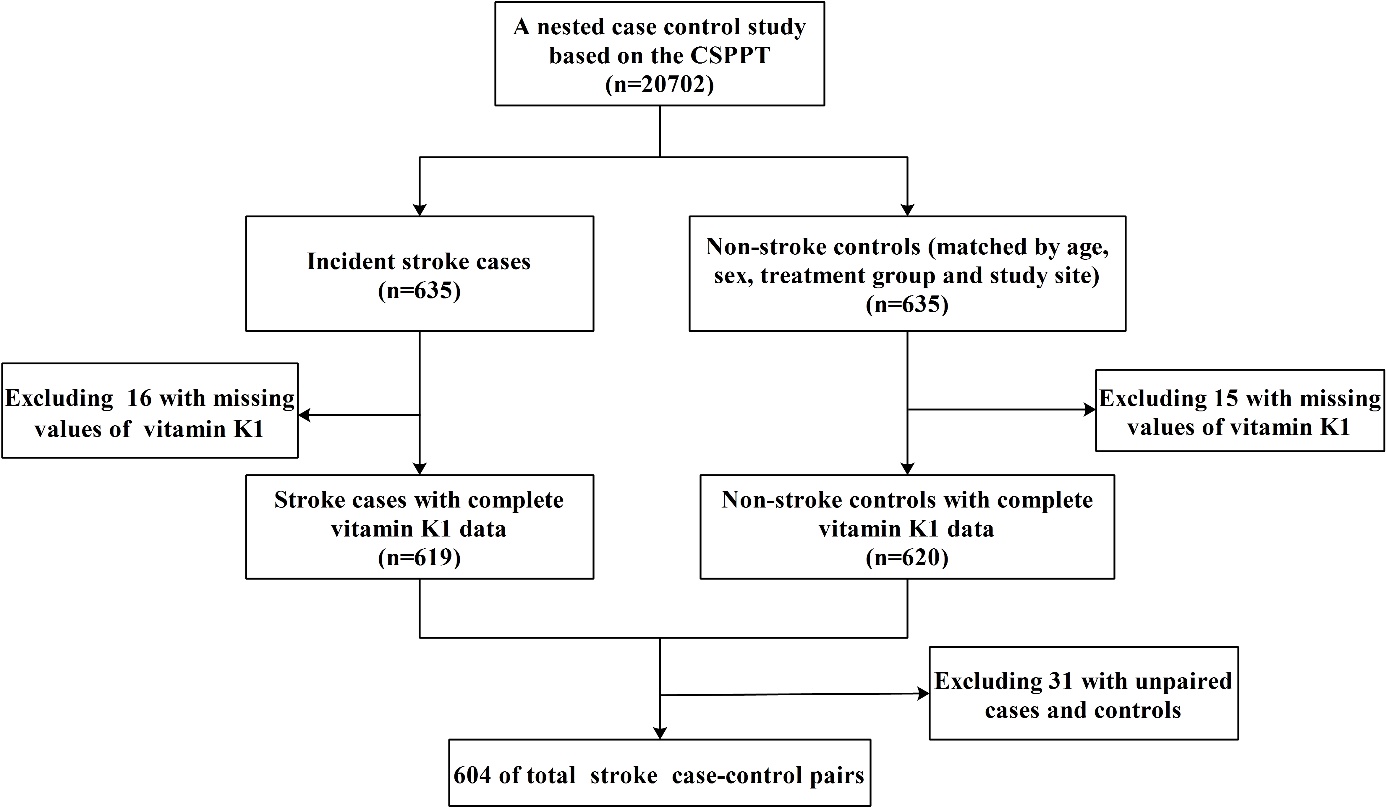 |
| --- |
| **Supplemental Figure 1** Flow chart of the study participants using a nested case–control design (CSPPT: China Stroke Primary Prevention Trial). |

| 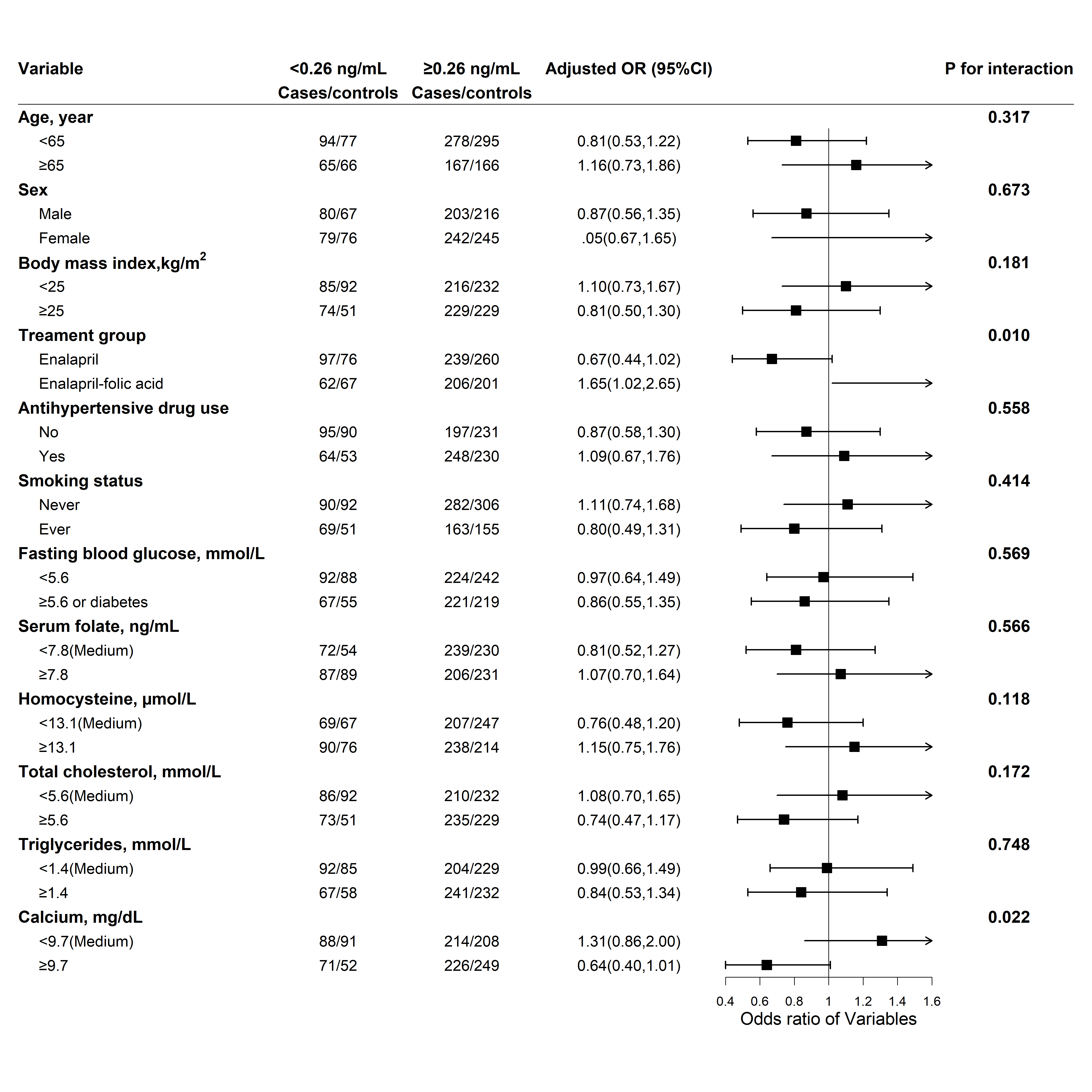 |
| --- |
| **Supplemental Figure 2** Forest plots on the association of plasma vitamin K1 with the risk of incident total stroke (adjusted OR and 95%CI), stratified by pertinent covariables. In addition to the matching factors (age, sex, treatment group, study site), the models adjusted for body mass index, baseline systolic blood pressure, time-averaged systolic blood pressure and diastolic blood pressure during treatment, baseline fasting blood glucose, total cholesterol, triglycerides, folate, antihypertensive treatment, **smoking status** and **antiplatelet drug usage** at baseline. CI: confidence interval; OR: odds ratio. |

| 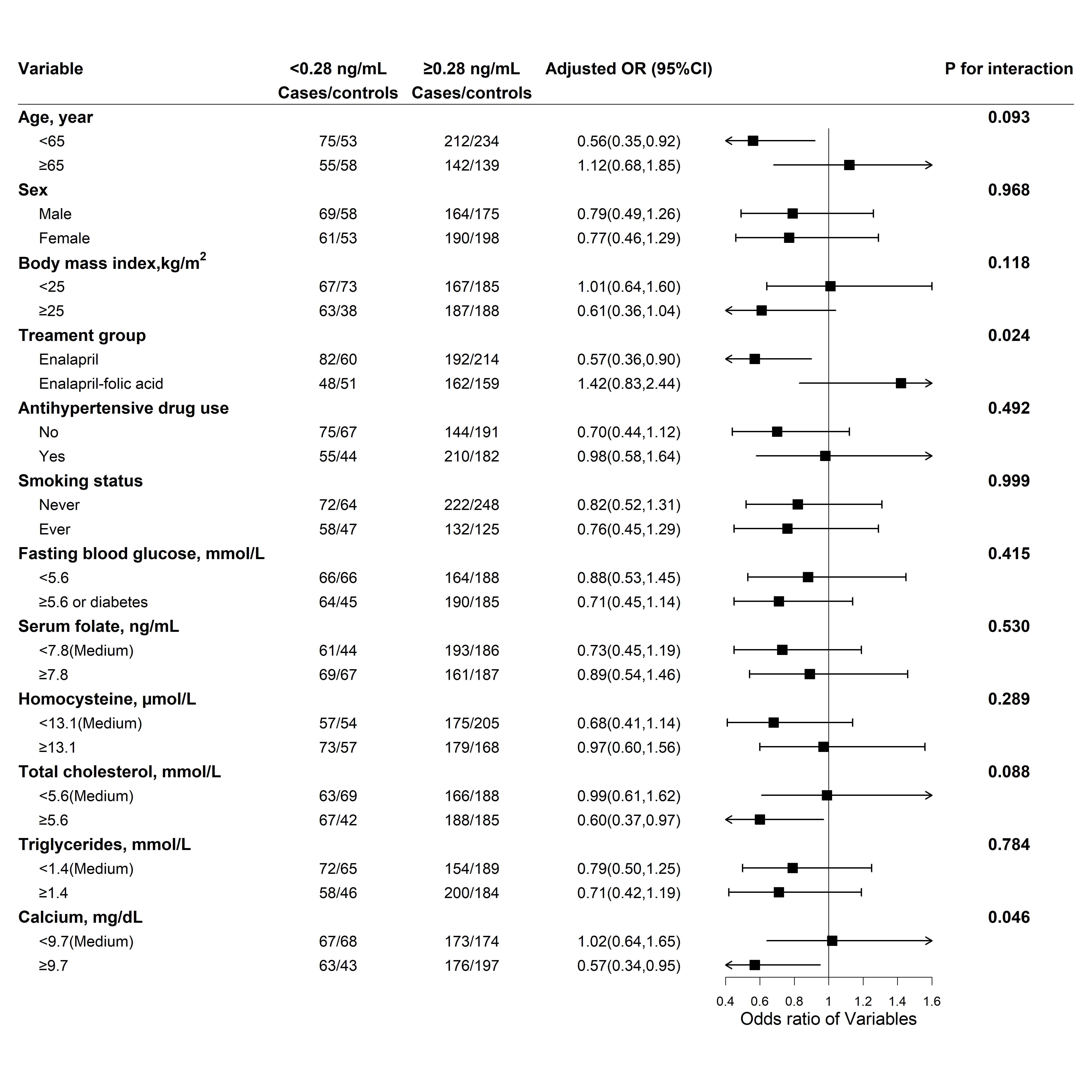 |
| --- |
| **Supplemental Figure 3** Forest plots on the association of plasma vitamin K1 with the risk of incident ischemic stroke (adjusted OR and 95%CI), stratified by pertinent covariables. In addition to the matching factors (age, sex, treatment group, study site), the models adjusted for body mass index, baseline systolic blood pressure, time-averaged systolic blood pressure and diastolic blood pressure during treatment, baseline fasting blood glucose, total cholesterol, triglycerides, folate, antihypertensive treatment, **smoking status** and **antiplatelet drug usage** at baseline. CI: confidence interval; OR: odds ratio. |
